# The suitability of structural soil for the development of trees growing in urban areas

**DOI:** 10.7717/peerj.20407

**Published:** 2026-02-20

**Authors:** Joanna Kosno-Jończy, Marzena Suchocka, Tatiana Swoczyna, Joanna Dudek-Klimiuk, Żaneta Tuchowska

**Affiliations:** 1 Institute of Environmental Engineering, Warsaw University of Life Sciences, Warsaw, Poland; 2 Institute of Horticultural Sciences, Warsaw University of Life Sciences, Warsaw, Poland; 3 Institute of Agriculture, Warsaw University of Life Sciences, Warsaw, Poland

**Keywords:** Urban trees, Stress factors, Site conditions, Sidewalk materials, Photosynthetic efficiency, *Tilia tomentosa* Moench

## Abstract

**Background:**

Tree survival in urbanized areas is increasingly challenged by dense city infrastructure, including underground systems. Engineered planting systems, such as structural substrates, offer more effective solutions for tree planting in urban sites where natural soil is unavailable. This study aims to assess the physiological responses of trees to different substrates in the context of long-term growth in modified urban habitats.

**Methods:**

Eight *Tilia tomentosa* Moench trees were planted in four substrate types: structural soil (SS), a mixture of crushed stone and soil; compacted soil (CS), simulating urban soil conditions; a mixture of soil and rubble with impermeable pavement (AS), representing harsh urban conditions; and natural soil (C), serving as the control. In the fifth and sixth years after planting, chlorophyll *a* fluorescence, relative chlorophyll content (Chl), and epidermal flavanols (Flv) were measured.

**Results:**

The highest values *F*_v_/*F*_m_ were observed in the control and SS samples (average *F*_v_/*F*_m_: 0.78–0.85), indicating good tree health, in contrast to the AS group. Energy dissipation per reaction center (DI_0_/RC) showed no significant differences between SS and C, except in September 2022. Indicators such as energy transfer into the electron transport chain (ET_0_/TR_0_), performance index (PI_ABS_), and chlorophyll content declined slightly in SS compared to C, but remained higher than in AS and similar to CS. Flavanol content was lower in both the control and SS groups, suggesting no evident stress response.

**Conclusion:**

This study confirms that structural soil provides favorable habitat conditions for urban trees, supporting undisturbed photosynthetic processes over the long term, comparable to natural soil. Structural substrates show strong potential as effective solutions for improving urban tree soil conditions, particularly in areas with soil compaction or limited root space beneath pavements. Future research should explore potential nutrient limitations, especially nitrogen availability, in structural soils.

## Introduction

Climate change, including more frequent and intense extreme events, has caused a wide range of negative impacts on the health and living conditions of people around the world, and this phenomenon is expected to intensify in the future (Kearl & Vogel, 2023). The crisis also correlates with the loss of biodiversity, including the decline and extinction of various species and ecosystems. It also correlates with pollution, including the contamination of air, water and soil with harmful substances (Woodward et al., 2023). A diversity of plant species play an essential regulatory role in urban biodiversity, as a habitat and shelter for other species (Fauk & Schneider, 2023). Trees facilitate carbon storage and sequestration, and removal of particulate matter from the environment (SO_2_ pollutants, NO_2_ contaminants, PM_10_, and PM_2.5_ pollutants) (Song et al., 2020; Suchocka et al., 2023). The urban heat island phenomenon is another important aspect. It can be defined as the difference in surface or air temperature between urban and rural areas. One reason for this phenomenon is the low density of vegetation which should provide shade and transpiration cooling (Frank & Backe, 2023). The above arguments show us that we should take care of the already existing trees in the cities by improving their living conditions, and to use solutions that allow the introduction of trees in places that are difficult to access.

Human population growth and the increasing demand for urban living space invariably exert pressure on the urbanized habitat (Blewett & Marzluff, 2005; McDonald, Kareiva & Forman, 2008; Nagendra & Gopal, 2010). Human activities result in a high proportion of paved surfaces, the fragmentation of biologically active areas, the construction of and renovation of the already existing dense underground infrastructure network, and the creation of impermeable surfaces that impede oxygen and water access to tree roots (Embrén et al., 2009; Stagoll et al., 2012; Czaja, Kołton & Muras, 2020). This as a result creates difficulties in expanding tree canopy cover, which is crucial for the wellbeing of the cities inhabitants (McGee et al., 2012; Ow & Ghosh, 2017a; Ow & Ghosh, 2017b; Konijnendijk, 2021a; Konijnendijk, 2021b; Nieuwenhuijsen et al., 2022; Ajmi et al., 2023). Unfavorable microclimatic conditions, *i.e.,* soil compaction, air and soil drought, reduced water retention in soil, soil pollution and salinity, are thought to bethe most important factors responsible for urban tree damage (Layman et al., 2016; Swoczyna & Latocha, 2016). Trees are observed to die or decline due to the surrounding urban development, mechanical damage and often a gradual weakening of their vitality (Vitali et al., 2019; Suchocka et al., 2021). This process particularly affects trees growing adjacent to pavement (Dmuchowski, Baczewska & Bragoszewska, 2011; North et al., 2017). Covering the root zone with pavement affects the infiltration rate, surface runoff and the water supply for trees (Blume, 2000; Bhaduri et al., 2001; Conway, 2007).

Engineer-driven tree planting solutions can address the problem of unfavorable urban conditions and the tree-infrastructure conflict, especially in the context of soil compaction and the problem of water access in root zones (Socash & Mayer, 2008; Ow & Ghosh, 2017a). One of the many solutions that can be applied is the use of structural soils (Bassuk, Grabosky & Trowbridge, 2005; Kociel et al., 2018), designed to provide additional space for the root system’s development under pavement. This can also be considered a solution for the soil exposed to compaction within the tree root systems (Grabosky et al., 1998; Ow & Ghosh, 2017a; Ow & Ghosh, 2017b). The modification of the structural soils near and under pavement represents a modality of environmental renovation that both supports the pavement and enables root growth (Shi et al., 2023). Structural soil is used to create a high porosity matrix that can be compacted to engineering load-bearing standards while retaining the physical properties conducive to aeration, hydration, and root elongation (Xiao & McPherson, 2008). Structural soil is composed of a mixture of substrate and crushed aggregate (a stone-soil mixture). The aggregate forms the framework that supports the pavement structure. Friction between the aggregate provides the bearing capacity of the subgrade (Bassuk et al., 1996). Even after it is compacted, space remains which provides room for root growth and root zone aeration (Trowbridge & Bassuk, 2004; Leake, 2007; Grabosky & Bassuk, 2016). Some of the space between the aggregate is filled with substrate, which provides the plant roots with oxygen, water and nutrients (Ow & Ghosh, 2017b).

A number of studies indicate that structural soils are more effective in enhancing habitat conditions and providing better growing conditions for urban trees than compacted urban soil (Grabosky & Bassuk, 1995; Grabosky et al., 1998; Grabosky, Bassuk & Marranca, 2002; Bühler, Kristoffersen & Larsen, 2007; Suchocka & Milanowska, 2013). They can be used both for tree planting and for improving the growth conditions of existing trees (Suchocka & Ziemiańska, 2013). This solution replaces the existing soil in the root systems of the trees and is used in the presence of signs of abiotic stress, resulting in tree vitality improvement (Alvem et al., 2009). Studies by other authors indicate that trees benefit from the use of structured substrates, primarily due to the moderate drainage this solution provides thanks to high porousness of the substrates which leads to higher transpiration (Ow & Ghosh, 2017a; Ow & Ghosh, 2017b). Results from studies conducted at Cornell University and the Urban Horticulture Institute have shown an increased growth of trees in structural soils, with better rooting, less susceptibility to toppling and a longer life expectancy on the urban sites (Socash & Mayer, 2008). More intense shoot growth and root proliferation were observed in trees planted in structural soils compared to trees growing in a standard sidewalk profile (Grabosky et al., 2001; Grabosky, Bassuk & Marranca, 2002; Bühler, Kristoffersen & Larsen, 2007). Research at Cornell University showed that tree roots in structural soil grow deep into the profile, up to about 91 cm, away from varying temperatures on the sidewalk surface. For this reason, the roots are less affected by the urban stress and by the damage caused by pavement repairs and reconstruction. Observations of trees planted in the McCarren Park in Brooklyn, NY indicated that 17 years after planting, the trees in structural soil experienced similar conditions to trees in the grass strip, and GPR data suggested that the tree roots had thoroughly colonized the structural soil profile (Bassuk et al., 2015). A study by Bartens, Wiseman & Smiley (2010) focused on the stability of two tree species in different soil types: natural (traditional tree pit), and suspended pavement design, as well as two types of stone mixes at 3 years after planting. In the case of *Ulmus parvifolia*, the soil type did not affect the stability of the plant. *Prunus serrulata* grown in the gravel/soil mix withstood nearly two times the trunk deflection force compared to trees planted in the other soil treatments, which might be related to the aforementioned length of root growth—it was up to 60 times longer in this type of soil. The physical characteristics of the gravel-based skeletal soil might contribute to tree stability because the stones are dense and angular therefore allowing them to interlock more effectively.

Urban habitat conditions are characterized by limited space available to the root system of trees, an impaired soil-air gas exchange (Balakina et al., 2005), inappropriate soil water properties (Qin, Li & Fu, 2013) and low nutrient abundance (Craul, 1992; Breś, 2008; Suchocka, 2011; Muüllerovaá, Vítková & Vítek, 2011). Water shortages are a major abiotic stress for trees in urban environments (Wang et al., 2020; Hannus et al., 2021). Due to water deficiency, trees growing near sidewalks show limited growth in height and a smaller basal diameter than their counterparts growing on grass or unpaved land (Chen et al., 2017; Moser et al., 2017; Ow, Ghosh & Yusof, 2019). Leaf and photosynthetic apparatus dysfunctions are also evident (Swoczyna et al., 2015; Kalaji et al., 2018; Mihaljević et al., 2021). Dehydrated leaf cells lose their ability to expand, and the closed stomata reduces photosynthetic efficiency (Cregg & Dix, 2001). Less drought-resistant species or varieties show a decrease *F*_v_/*F*_m_ under drought stress (Percival & Sheriffs, 2002; Percival, Keary & AL-Habsi, 2006; Faraloni et al., 2011). In numerous experiments, the increased heat dissipation of excess energy is the first symptom of drought stress (Guha, Sengupta & Reddy, 2013; Lee et al., 2016). This may result in diminished electron transport towards end electron acceptors which provides energy for the Calvin-Benson cycle (Guha, Sengupta & Reddy, 2013; Kalaji et al., 2018). In some cases, drought stress influenced the functioning of the oxygen evolving complex at the donor side of the photosystem II (Guha, Sengupta & Reddy, 2013; Kalaji et al., 2018; Mihaljević et al., 2021) and the pool of active reaction centers (Guadagno, Beverly & Ewers, 2021).

A fast, non-invasive method for detecting disorders of the photosynthetic apparatus at a cellular level is to measure chlorophyll *a* fluorescence with a high-time resolution fluorimeter after the dark adaptation of the samples (Kalaji et al., 2014; Swoczyna et al., 2022; Sieczko et al., 2022). This method makes it possible to detect changes in the photosynthetic process caused by biotic and abiotic stress even before the appearance of visible symptoms of health deterioration, *i.e.,* leaf chlorosis, necrosis, or growth restriction (Naumann, Young & Anderson, 2008; Swoczyna et al., 2010). Light absorbed by the photosynthetic apparatus in chloroplasts is used for photochemical processes, dissipated as heat and re-emitted as fluorescence. The analysis of the fluorescence signal enables the assessment of the relationships between these processes (Maxwell & Johnson, 2000). The so-called prompt fluorescence technique requires the adaptation of a sample to darkness which inactivates all the photochemical processes. Following dark adaptation, a rapid illumination of the sample with a saturating pulse of light is performed and then the prompt chlorophyll *a* fluorescence rise gives a picture of the processes inside and around the photosystem II (Strasser, Tsimilli-Michael & Srivastava, 2004). Changes during the initial rise of the fluorescence signal (*i.e.,* until the fluorescence yield reaches its maximum value) can be primarily correlated with the events taking place in the course of the successive reduction of the electron acceptors of the entire photosynthetic electron transport chain (Stirbet & Govindjee, 2011). The so-called JIP-test, derived from the calculations of the fluorescence yield at consecutive steps of the fluorescence rise, allows for an assessment of stress influence on the examined plants (Percival, Keary & AL-Habsi, 2006; Bussotti et al., 2010; Swoczyna et al., 2020). Moreover, the analysis of the fluorescence transient enables an insight into the particular phenomena involved in the light energy conversion (activity of reaction centers, electron transport beyond photosystem II *etc.*).

Typically, the content of flavanols in plants increases under stress conditions (Havaux & Kloppstech, 2001). The simple process of UV irradiation itself increases the concentration of flavanols (Warren et al., 2002). Flavanols are also formed due to the interference of a pathogenic agent, injury, mechanical pressure, water deficit or osmotic and salt stress (Zhuang et al., 2023). Different types of environmental stress (water deficiency, flooding, nutrient deficiency, salt stress, *etc.*) affect particular photochemical processes (Naumann, Young & Anderson, 2008; Pollastrini et al., 2014; Kalaji et al., 2018; Swoczyna et al., 2019; Sieczko et al., 2022), thus, the chlorophyll *a* fluorescence technique coupled with the analysis of the chlorophyll and epidermal flavanols content can give an overall assessment of the trees’ photosynthetic response to soil conditions.

The solutions based on structural substrates, used as a way to improve unfavorable soil conditions for tree growth in urbanized public spaces, have been relatively rare up to now. Literature lacks studies of the long-term response of trees to the application of this solution, and our research fills this niche. It was hypothesized that the structural substrate provides a significant improvement in habitat conditions for tree development in urban environments over a multi-year period. The aim of the research is to assess physiological responses in the context of long-term tree growth in a modified habitat, to answer the question of whether and how structural soil affects the habitat conditions of trees, 5 and 6 years after the application of this solution.

## Materials & Methods

### Site location and experimental design

**Figure 1:**
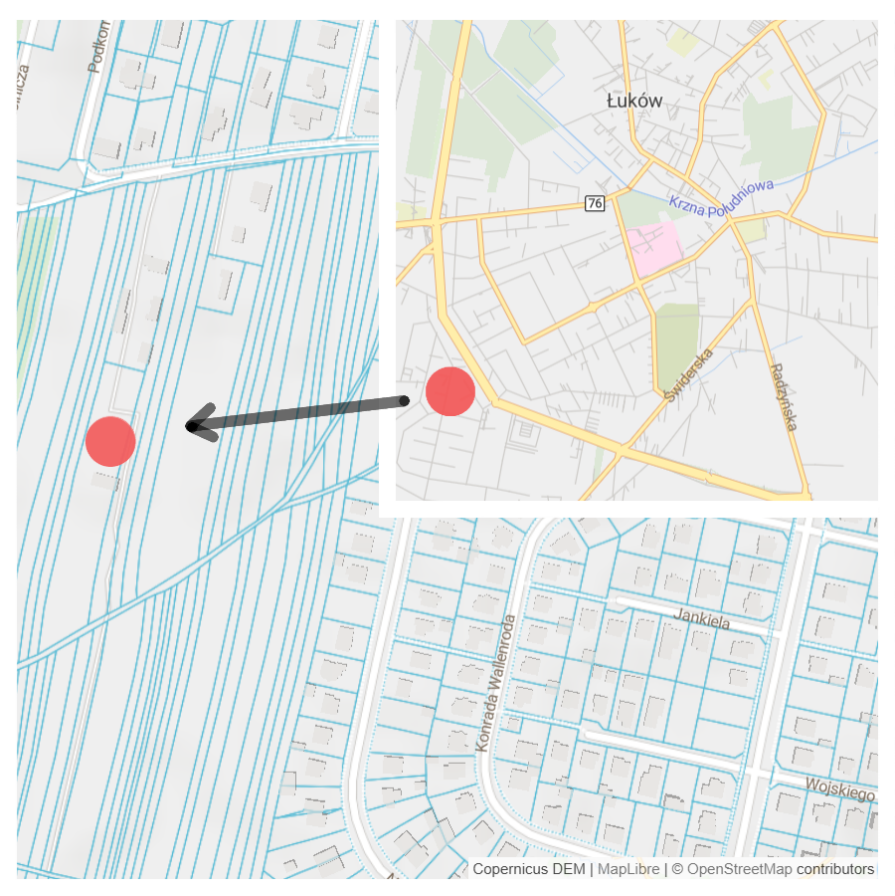
Location of the experimental plot, Source: https://www.openstreetmap.org/.

**Figure 2:**
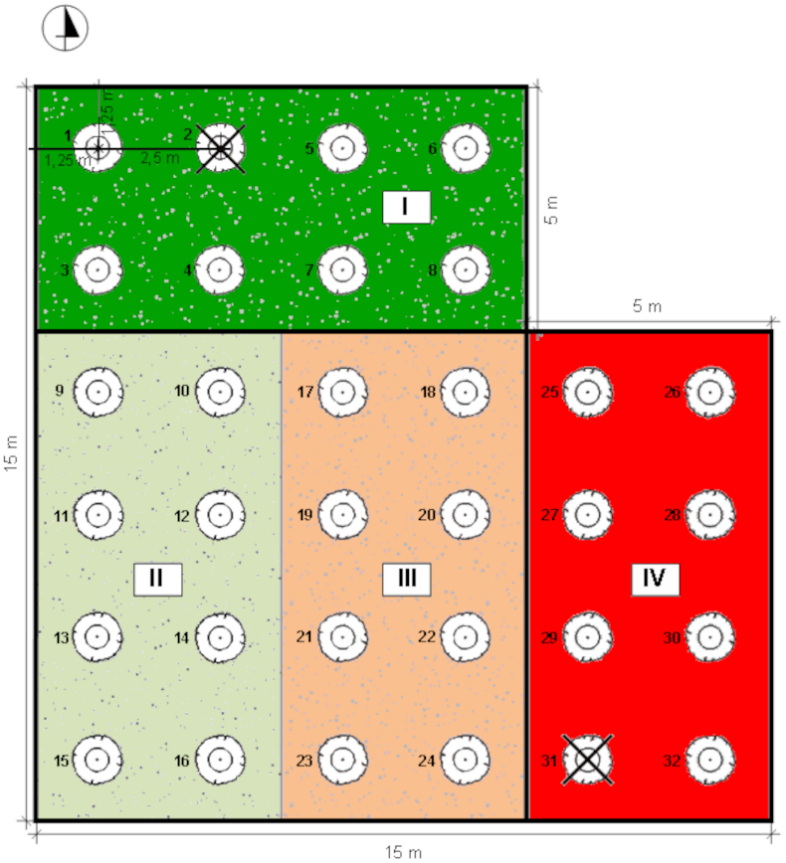
Distribution of individual sectors of the experimental plot. I: SS Structural soil, II: C Control, III: CS Compacted soil, IV: AS Impermeable paving; 1-32: number of trees.

The experiment was conducted on Rolnicza street in Łuków, Poland (51°55′44″N, 22°22′46″E) (Fig. 1). Four experimental plots, each of an area of 5  × 10 m, isolated from each other with vertical root barriers (of a depth of one m) were prepared. The root barriers (Mullaney, Lucke & Trueman, 2015) were used to avoid rainwater and root penetration from the neighbouring soil volume. The soil filling of the plots was selected as follows: (1) structural soil (SS), with permeability parameters appropriate for the proper development of trees, meeting the requirements of load-bearing capacity and nutrient content. The structural soil was covered with a permeable surface made of resin concrete; (2) natural soil as a control, without interfering with the ground (C); (3) soil compacted up to 1.4 g cm^−3^ (McGrath & Henry, 2016) hereinafter referred to as ‘compacted soil’ (CS); (4) a mixture of soil and rubble covered with impermeable concrete slabs (Kociel, 2018), simulating extremely harsh urban habitat conditions (AS) (Fig. 2).

**Figure 3:**
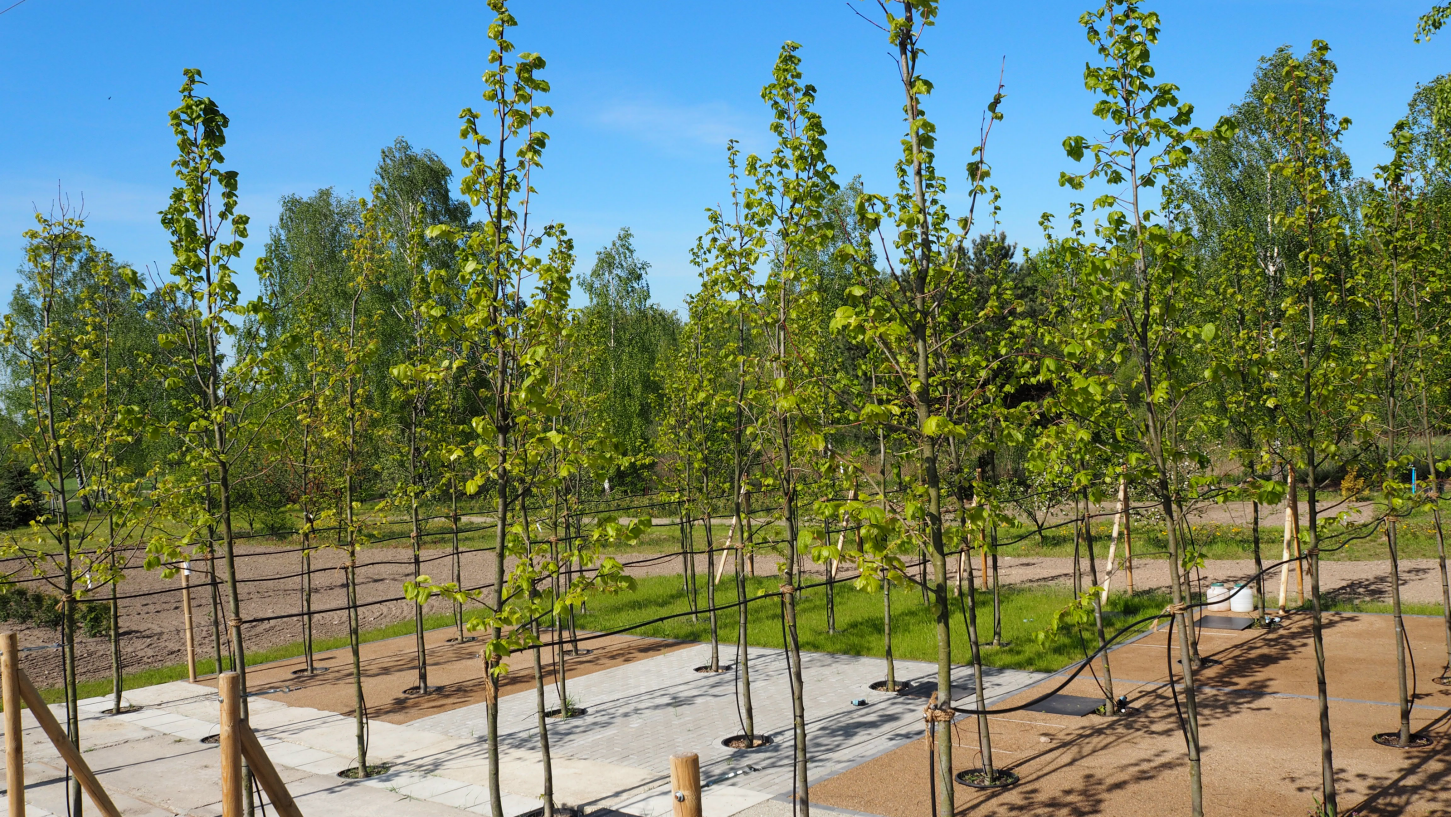
View of the experimental plot, May 2017.

In the spring of 2016, eight young specimens of the *Tilia tomentosa* Moench. were planted on each plot (trunk girth: 10–12 cm) (Figs. 3 4). For the first 3 years, the plants were watered using drip irrigation. The trees had not been fertilized since planting and were not watered since 2019. The experimental plot was not shaded, and the trees grew in equal microclimate conditions. In 2021 two specimens were excluded from the measurements due to a mechanical injury within the crown, due to weather conditions—a strong wind. Ultimately, a total of seven, eight, eight and seven specimens planted in SS, C, CS and AS, respectively, were used for the photosynthetic parameter’s test.

**Figure 4:**
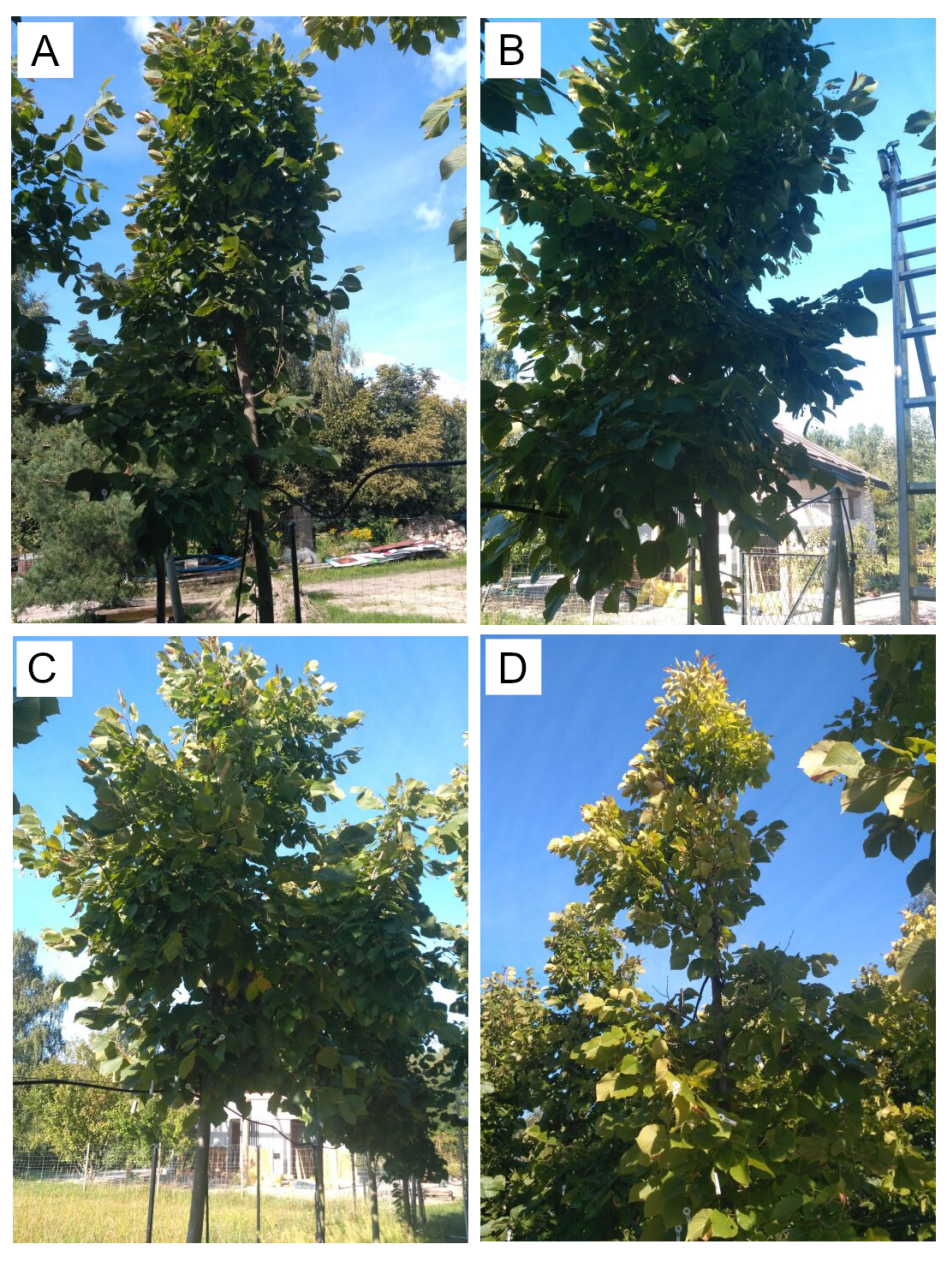
Studied trees growing on experimental plot, September 2021. (A) tree No. 1: (SS, structural soil); (B) tree No. 15: (C, control); (C) tree No. 24: (CS, compacted soil); (D) tree No. 29: (AS, impermeable paving).

### Structural soil content and preparation

The experimental pavement sub-base (SS) was built of a 63–120 mm coarse crushed stone aggregate (aeration layer—40 cm thick), and a 31.5–63 mm crushed stone aggregate (vegetation layer—20 cm thick) installed on the first layer. Each of the two layers was compacted successively. The spaces between the previously compacted crash stone layers were filled with substrate. Organic substrate (a mixture of leaf composts, NPK mineral fertilizer, pH 5.0−6.5 and native soil at pH 7) was washed into both layers successively. The compaction of the stone frame ensured a load-bearing capacity (the bearing capacity of the top layer equaled to E2 = 132 MPa), and the selection of the aggregate fractions ensured a uniform structure with high porosity after compaction. Aggregate was the load-bearing element of the system, and its particles met the durability and strength standards of grains provided for road structures (compressive strength and full frost resistance). The structural soil was characterized by the presence of air pockets, providing aeration and space for proper root development (Kociel, 2018).

### Climatic conditions

Meteorological data, *i.e.,* mean daily temperature and daily precipitation, were obtained from the Meteo Station of Siedlce (53°48′18″N, 21°38′27″E). Illuminance and air humidity were measured using a DHT22 sensor (Aosong Electronics Co., Ltd, Guangzhou, China). The data was obtained from *Twój Świat Jacek Mojski*, (51°55′29.422″N, 22°21′25.783″E) in Łuków, Poland.

### Physiological measurements

Chlorophyll *a* fluorescence was measured using a *HandyPEA* fluorimeter (Hansatech Instruments Ltd., King’s Lynn, Norfolk, Great Britain). A total of 54 measurements were taken for each tree, giving a total of 1,620 samples tested. The measurements of chlorophyll *a* fluorescence were carried out on July 6, August 7 and September 12 in 2021 and on June 2, August 5 and September 8 one year later in 2022. The measurements were taken between 10:00 a.m. and 12:00, on single leaves selected from the top, middle and bottom parts of the tree crown. A total of nine leaves were selected from each tree—three leaves were taken from three different heights—the lower, middle and upper part of the shaded part of the crown. The measurements were conducted on leaves while still attached to the plant. The leaves were dark-adapted using light-excluding clips which lasted for at least 20 min. The dark-adapted leaf samples were afterwards illuminated with 660 nm light of 3,500 µmol m^−2^s^−1^. In order to assess the photosynthetic efficiency of the trees the following parameters were analysed: *F*_v_/*F*_m_ expressing the fraction of the total energy flux trapped by the photosystem II reaction centers (PSII RCs), the probability of the electron movement from the first acceptor Q_A_ into the electron transport chain (*ψ*_Eo_ = ET_0_/TR_0_), the probability that an electron is transported to the electron acceptor side at PSI (*δ*_Ro_ = RE_0_/ET_0_), the amplitude of the I–P phase of the OJIP fluorescence transient (ΔV_IP_), the Performance Index on absorption basis (PI_ABS_), the ratio of variable fluorescence at 300 µs to variable fluorescence at 2 ms showing limitations in the donor side (at oxygen-evolving complex) of the photosystem II (V_K_/V_J_), effective dissipation per active reaction centre (RC) (DI_0_/RC), the rate of electron transport per active RC (ET_0_/RC) and the density of active reaction centres (RCs) per cross-section at point 0 (RC/CS_0_) (for detailed explanations see Strasser, Tsimilli-Michael & Srivastava, 2004; Salvatori et al., 2015; Bussotti et al., 2020).

The measurements of the relative chlorophyll content and leaf epidermal flavanols were conducted using a leaf-clip-type sensor Dualex^®^ (FORCE-A, Orsay Cedex, France). A total of 27 measurements were taken for each tree, giving a total of 810 samples tested. The measurements were carried out on July 6, August 7 and September 12 in 2021. They were taken between 10:00 a.m. and 12:00 on the other leaves along with fluorescence measurements (which were taken on nine leaves per tree—three leaves each at three levels—the lower, middle and upper part of the crown). The sensor enables non-destructive measurements (Cerovic et al., 2015).

### Statistical analyses

As the data showed no normal distribution (according to the Shapiro–Wilk test), the non-parametric Kruskall–Wallis and Dunn–Bonferroni *post-hoc* tests were used for the assessment of the significant differences between different habitat conditions. All the calculations were made using the STATISTICA version 13.0 software (TIBCO Software Inc., 2017, Palo Alto, CA, USA).

## Results

### Climatic Conditions

**Figure 5:**
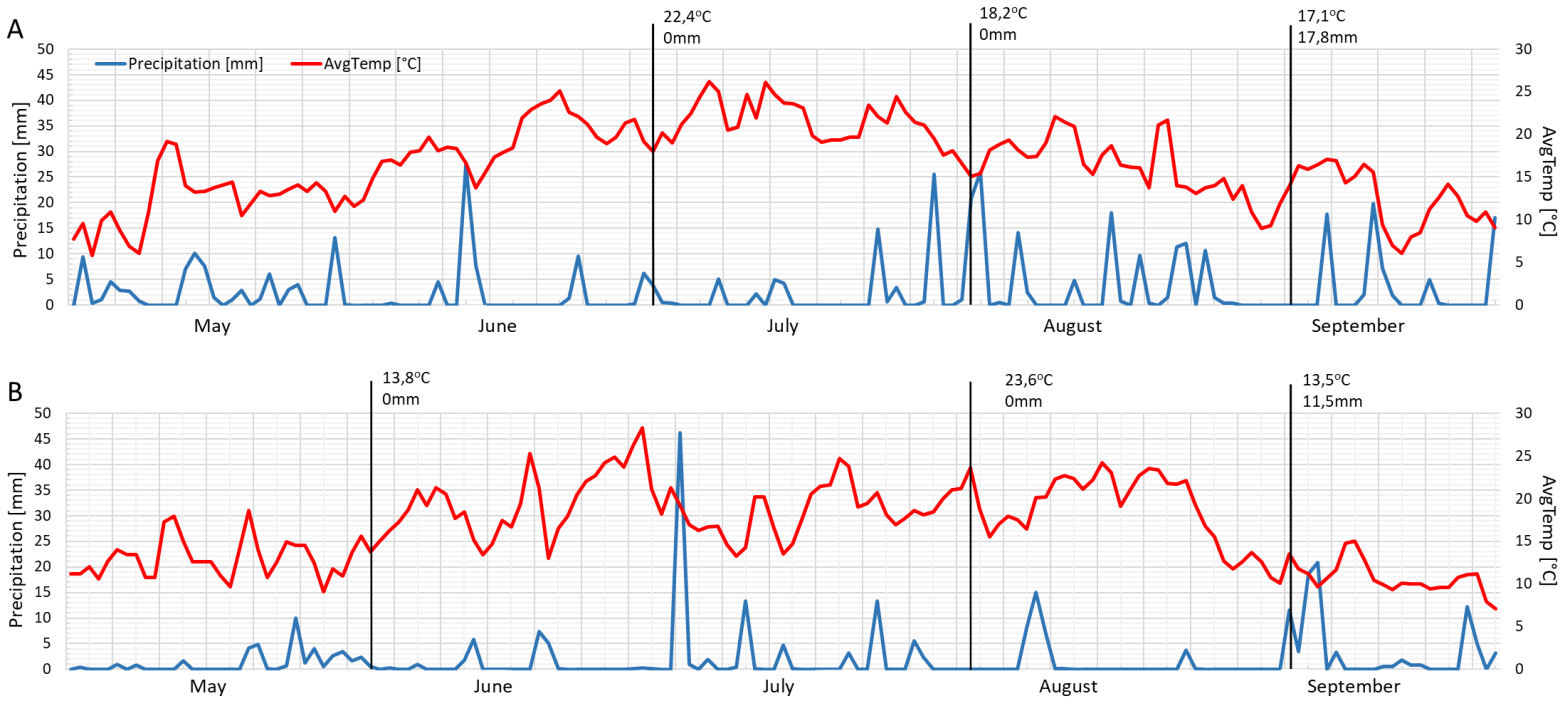
Mean daily temperatures and precipitation in May, June, July, August and September 2021 (A) and 2022 (B). Black lines show the measurement days.

The mean daily temperatures on the measurement days in 2021 were similar to each other (June 22.4 °C, August 18.2 °C and September 17.1 °C) (Fig. 5A). The months of June and September in 2022 were cooler (about 13 °C), while August was warmer than the previous year (23.6 °C) (Fig. 5B). The year 2021 was wetter, especially in August and September (Fig. 5A), while 2022 had a drier mid-summer (Fig. 5B). The measurements were performed following precipitation of 17.8 mm and 11.5 mm in September 2021 and September 2022, respectively (Figs. 5A–5B).

**Figure 6:**
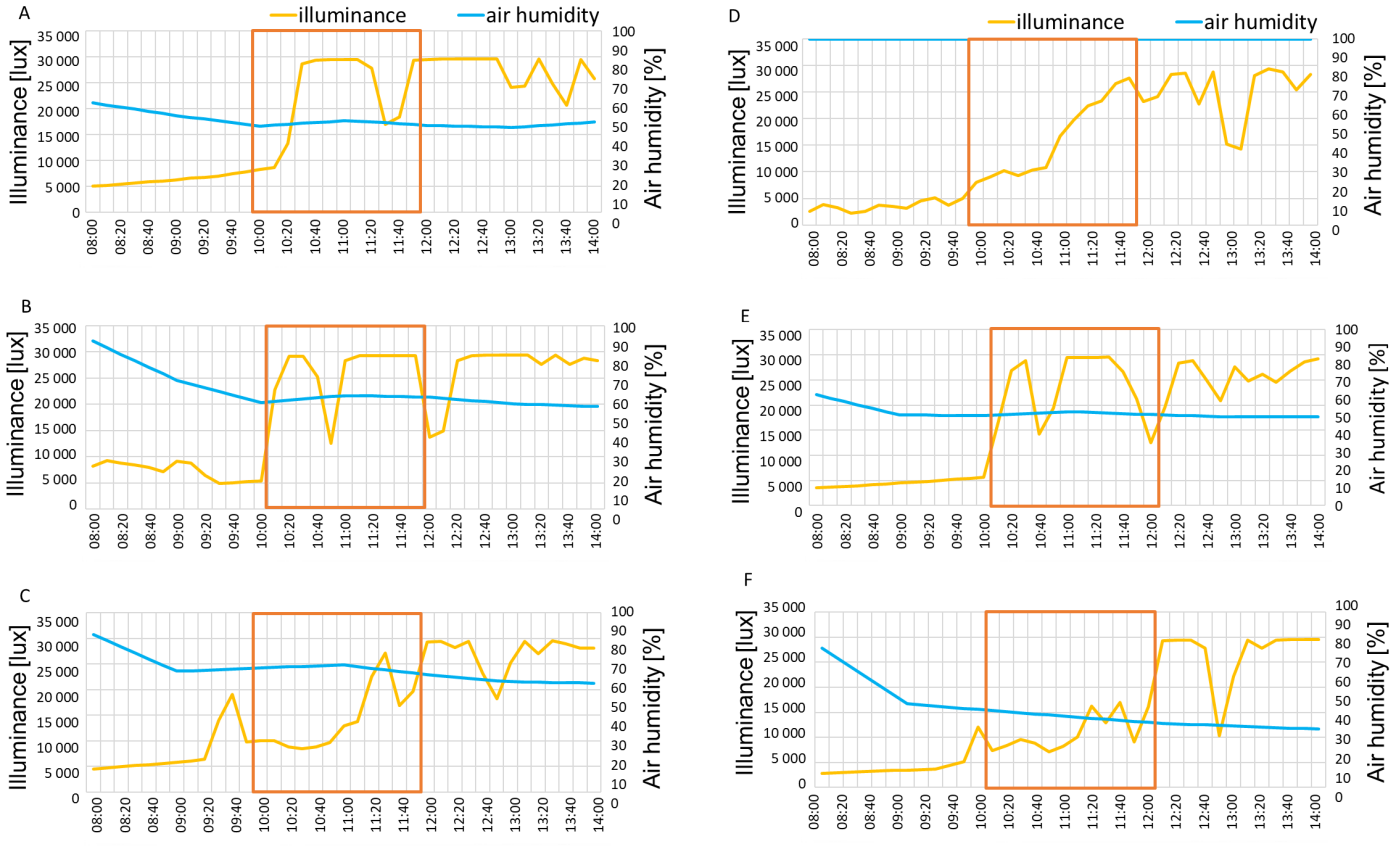
Illuminance and air humidity in measurement days in 2021: July 6 (A), August 7 (B), September 12 (C) and 2022: June 2 (D), August 5 (E), September 8 (F). Red lines show the measurement hours.

The illuminance in June and August of 2021 reached 30,000 lux at measurement hours, while in September higher values appeared only around 12 p.m (Figs. 6A–6C). In August 2022 illumination reached 30,000 lux while in June and September the morning values were approximately around 10,000 lux, gradually increasing to 25,000–30,000 lux by 12 p.m (Figs. 6D–6F). The air humidity percentage was around 50% in July, 60% in August and 70% September in the year 2021 (Figs. 6A–6C) and 100%, 50% and 40% in June, August and September 2022, respectively (Figs. 6D–6F).

### Chlorophyll fluorescence

**Figure 7:**
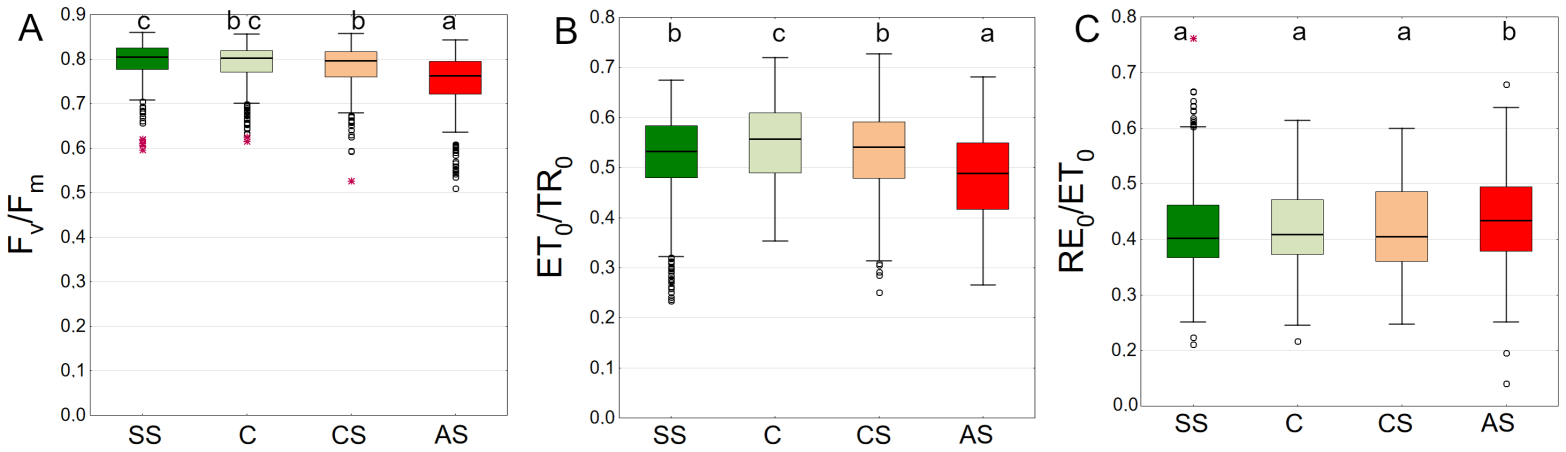
(A–C) *F*_*v*_/*F*_*m*_, *ψ*_*Eo*_ = *ET*_0_/*TR*_0_ and *δ*_*Ro*_ = *RE*_0_/*ET*_0_ parameters, results from all measurement dates. SS, structural soil; C, control; CS, compacted soil; AS, impermeable paving. Box-whiskers plots show medians and interquartile ranges (Q1–Q3 boxes), and outliers (circles), the whiskers extend to a max. of 1.5 times the interquartile range. Significant differences (*P* < 0.05) between the soil types are indicated by lowercase letters.

*F*_v_/*F*_m_ summarized from all measurement dates was the highest in SS and C with medians of 0.805 and 0.802, respectively. There was no difference between these plots. In CS and AS the *F*_v_/*F*_m_ differed significantly, with medians of 0.796 and 0.763, respectively (Fig. 7A). The leaves of trees growing in C showed the highest ET_0_/TR_0_ values compared to the other sectors. The difference was statistically significant. SS and CS showed similar levels (Fig. 7B). The differences for the RE_0_/ET_0_ were the least pronounced. Only the AS sector showed higher statistically significant values (Fig. 7C).

**Figure 8:**
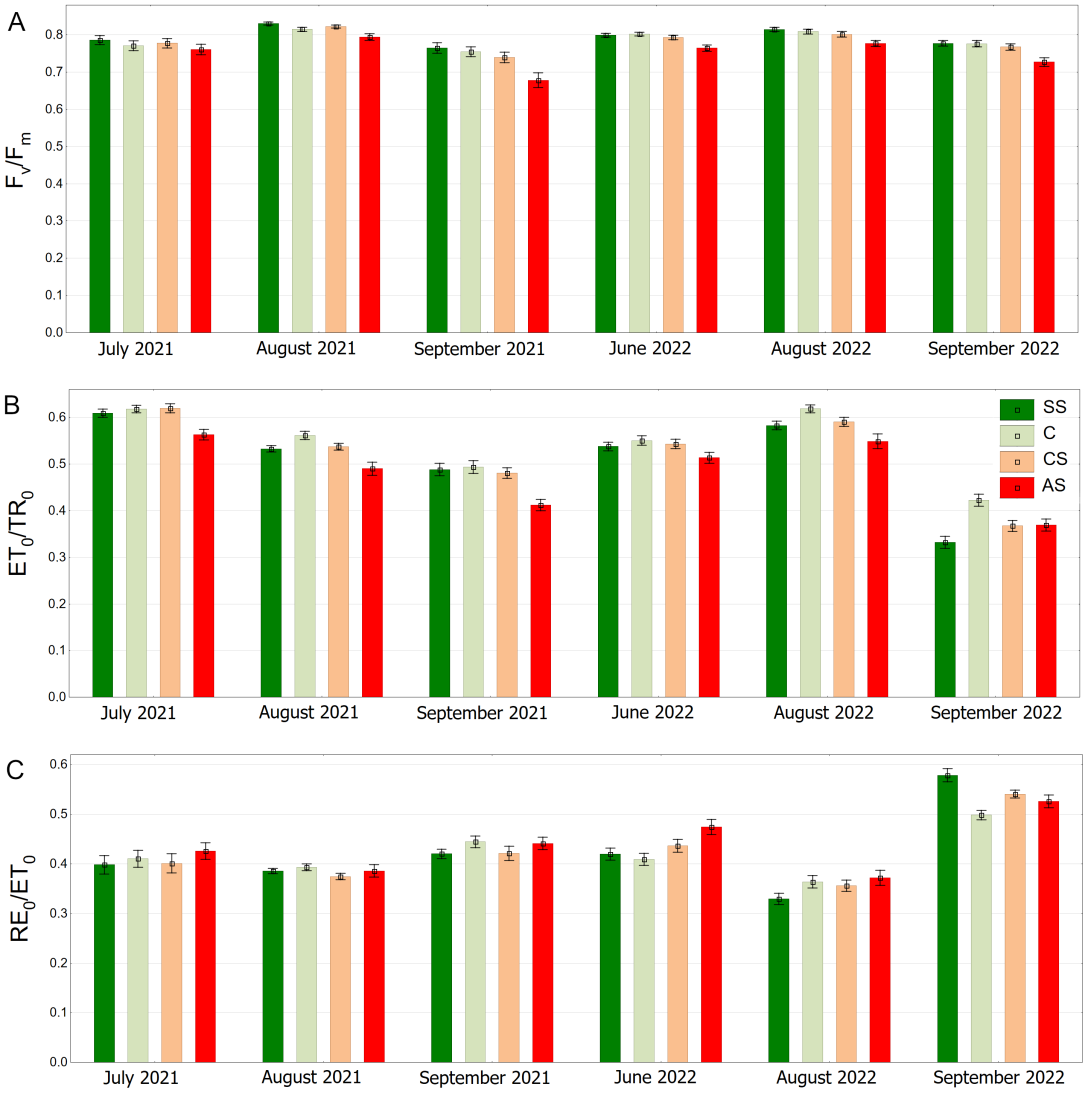
(A–C) *F*_*v*_/*F*_*m*_*ψ*_*Eo*_ = *ET*_0_/*TR*_0_ and *δ*_*Ro*_ = *RE*_0_/*ET*_0_ parameters, results from all measurement dates. SS, structural soil; C, control; CS, compacted soil; AS, impermeable paving. Bars show means and SE.

In the summer months (June, July and August) the *F*_v_/*F*_m_ medians were equal to or exceeded the value of 0.800. In September (2021 and 2022) *F*_v_/*F*_m_ in C, SS and CS tended to decrease. The trees surrounded by impermeable pavement showed lower *F*_v_/*F*_m_ values on each measurement day. That trend was particularly visible in September (Fig. 8A). As for the ET_0_/TR_0_ parameter similarly to the *F*_v_/*F*_m_, SS and C had higher values than AS, which was especially evident in September 2022. In August 2022, all groups reached relatively high values, but the values in AS were still the highest (Fig. 8B). RE_0_/ET_0_ values were similar in all groups. In addition a particularly noticeable increase in RE_0_/ET_0_ in September 2022 for SS relative to other sectors was found. In that month, the results for all sectors reached the highest levels (Fig. 8C).

**Figure 9:**
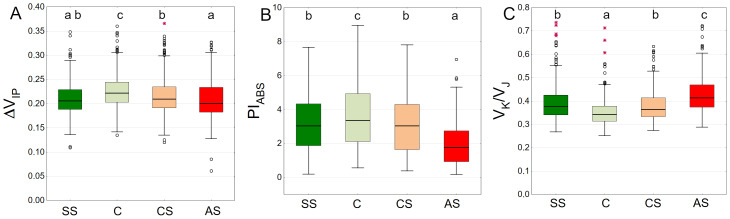
(A–C) Δ*V*_*IP*_, *PI*_*ABS*_ and *V*_*K*_/*V*_*J*_ parameters, results from all measurement dates. SS, structural soil; C, control; CS, compacted soil; AS, impermeable paving. Box-whiskers plots show medians and interquartile ranges (Q1–Q3 boxes), and outliers (circles), the whiskers extend to a max. of 1.5 times the interquartile range. Significant differences (*P* < 0.05) between the soil types are indicated by lowercase letters.

The highest values of the ΔV_IP_ index were observed in the control sector with a statistically significant difference. The lowest results were obtained by AS, SS and CS, respectively (Fig. 9A). The trees growing in the natural soil showed the highest values of PI_ABS_. The impervious pavement was responsible for the lowest values and there was no significant difference between the structural and compacted soil (Fig. 9B). The control plot showed statistically significant lowest values of V_K_/V_J_. The sector simulating extremely harsh urban habitat conditions had the highest value, while SS and CS reached intermediate values (Fig. 9C).

**Figure 10:**
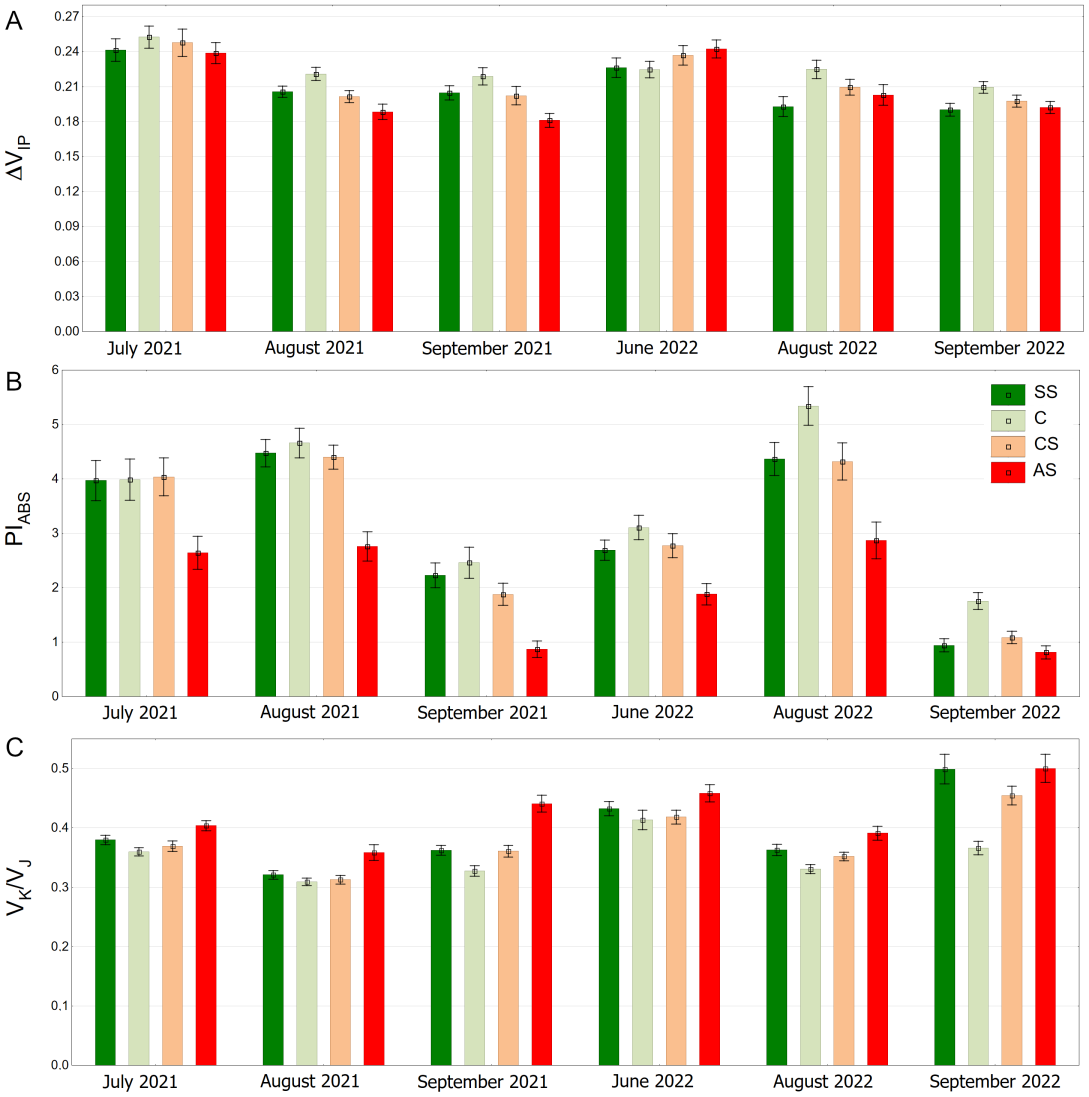
(A–C) Δ*V*_*IP*_, *PI*_*ABS*_ and *V*_*K*_/*V*_*J*_ parameters, results from all measurement dates. SS, structural soil; C, control; CS, compacted soil; AS, impermeable paving. Bars show means and SE.

The distribution of the ΔV_IP_ results is clearly seasonal. In September 2021 and September 2022, overall values are generally lower (Fig. 10A). The pattern of differences between the experimental plots was similar on all measurement days for the PI_ABS_ index. However, the results differed to some extent between months, showing the highest values in August and the lowest in September in both growing seasons (Fig. 10B). The AS variant had the highest V_K_/V_J_ values, especially in June 2022 and September 2022. August 2021 and August 2022 show lower values for all substrate types. (Fig. 10C).

**Figure 11:**
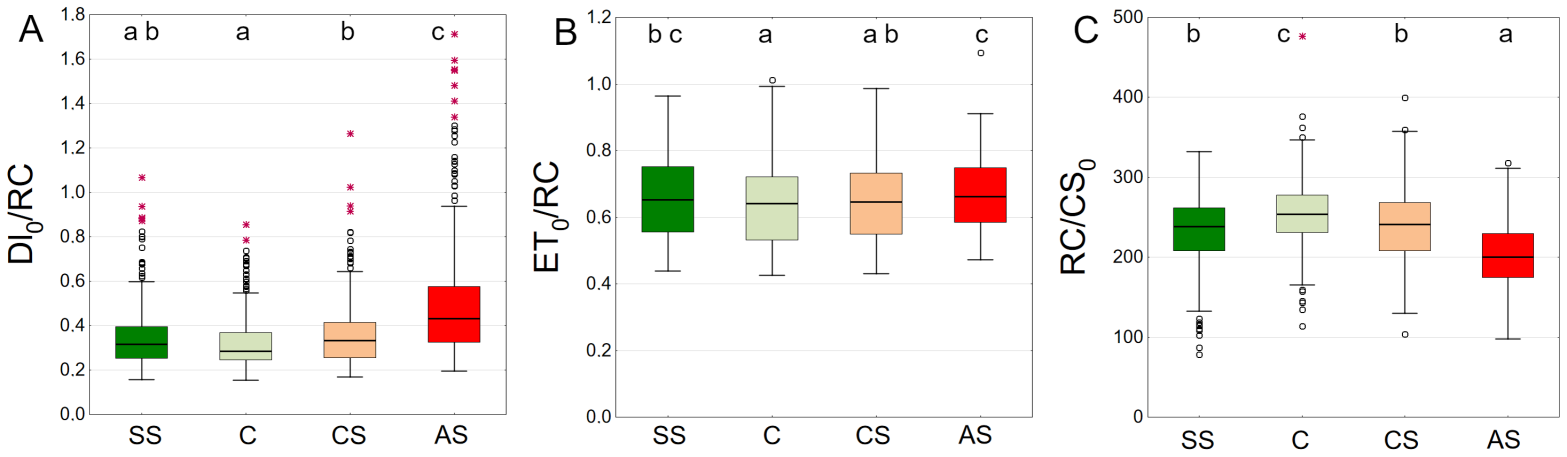
(A–C) *DI*_0_/*RC*, *ET*_0_/*RC* and *RC*/*CS*_0_ parameters, results from all measurement dates. SS, structural soil; C, control; CS, compacted soil; AS, impermeable paving. Box-whiskers plots show medians and interquartile ranges (Q1–Q3 boxes), and outliers (circles), the whiskers extend to a max. of 1.5 times the interquartile range. Significant differences (*P* < 0.05) between the soil types are indicated by lowercase letters.

Trees growing in control and structural soil had the lowest Dl_0_/RC values and did not significantly differ from each other. The AS variant clearly showed the highest values (Fig. 11A). C and CS show the highest median of ET_0_/RC while AS showed the lowest. The SS variant was in between (Fig. 11B). The highest RC/CS_0_ was found in C, followed by SS, CS and AS. There parameters did not significantly differ between SS and CS (Fig. 11C).

**Figure 12:**
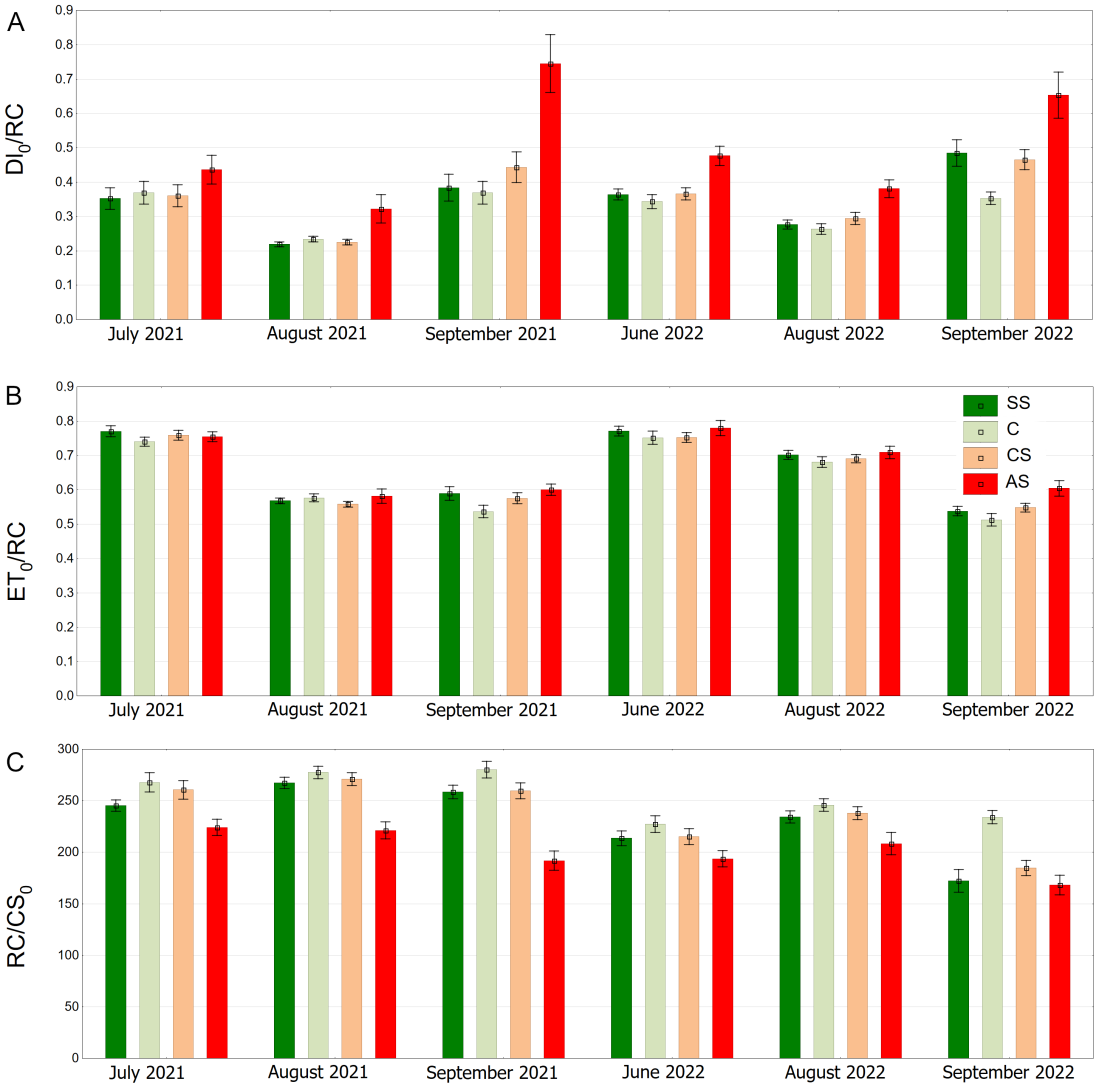
(A–C) *DI*_0_/*RC*, *ET*_0_/*RC* and *RC*/*CS*_0_ parameters, results from all measurement dates. SS, structural soil; C, control; CS, compacted soil; AS, impermeable paving. Bars show means and SE.

The AS variant showed the highest DI_0_/RC values, especially in September 2021 and September 2022. The SS, C and CS variants tended to have lower values, which were more stable over time. In August 2021, DI_0_/RC was the lowest for all plots (Fig. 12A). The highest ET_0_/RC values were observed in June 2022 and the lowest values occurred in September 2022—for all variants, especially SS and C (Fig. 12B). SS and C regularly showed the highest RC/CS_0_ values, especially in August and September of 2021. In September 2022, there was a sharp decline for all variants (Fig. 12C).

### Relative content of chlorophyll and epidermal flavanols

**Figure 13:**
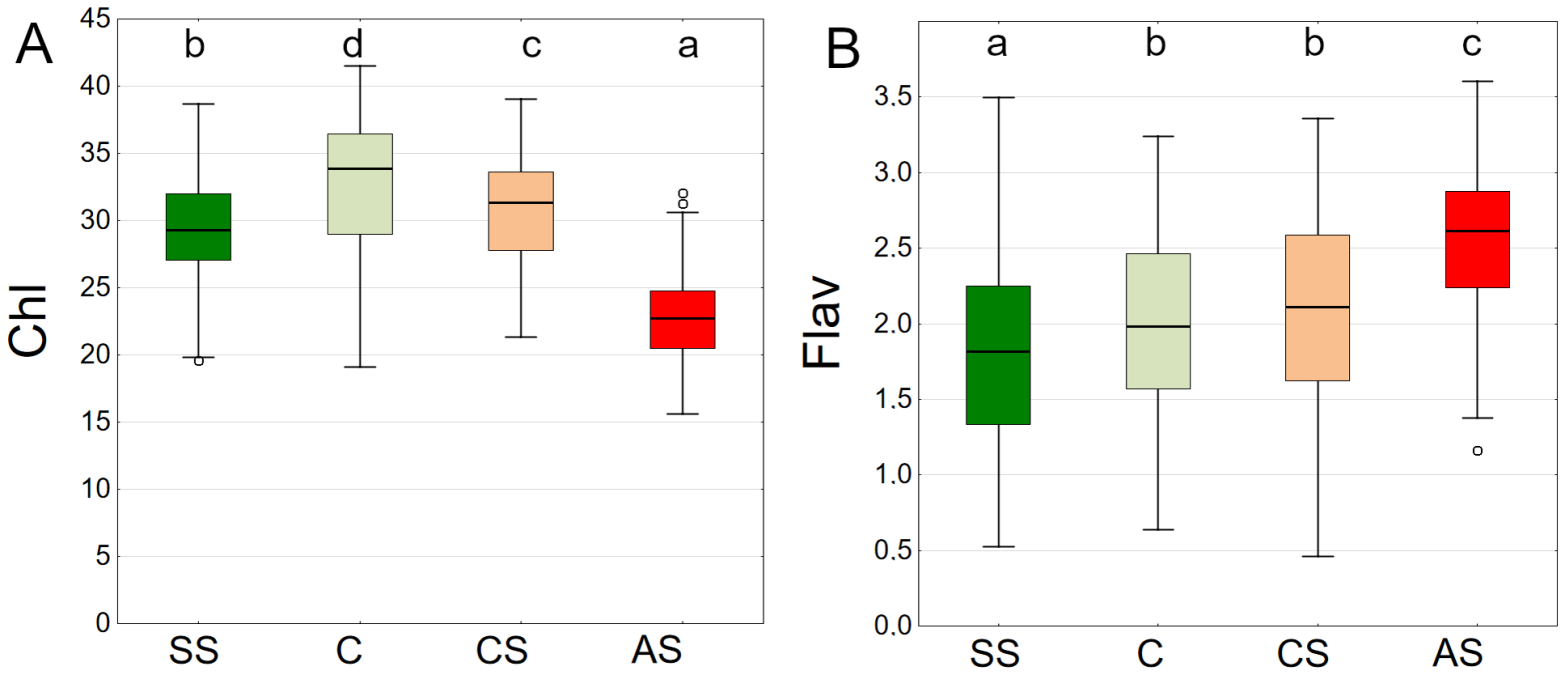
(A–B) Chlorophyll (Chl) and flavanols (Flav), results from all measurement dates. SS, structural soil; C, control; CS, compacted soil; AS, impermeable paving. Box-whiskers plots show medians and interquartile ranges (Q1–Q3 boxes), and outliers (circles), the whiskers extend to a max. of 1.5 times the interquartile range. Significant differences (*P* < 0.05) between the soil types are indicated by lowercase letters.

The results summarised from all dates showed the highest chlorophyll content in C, followed by CS, SS and AS. The differences between plots were significant (Fig. 13A).

The lowest values of epidermal flavanols were found in SS, followed by C, CS and AS The results were statistically significant, with no statistical relationship found only between C and CS (Fig. 13B).

**Figure 14:**
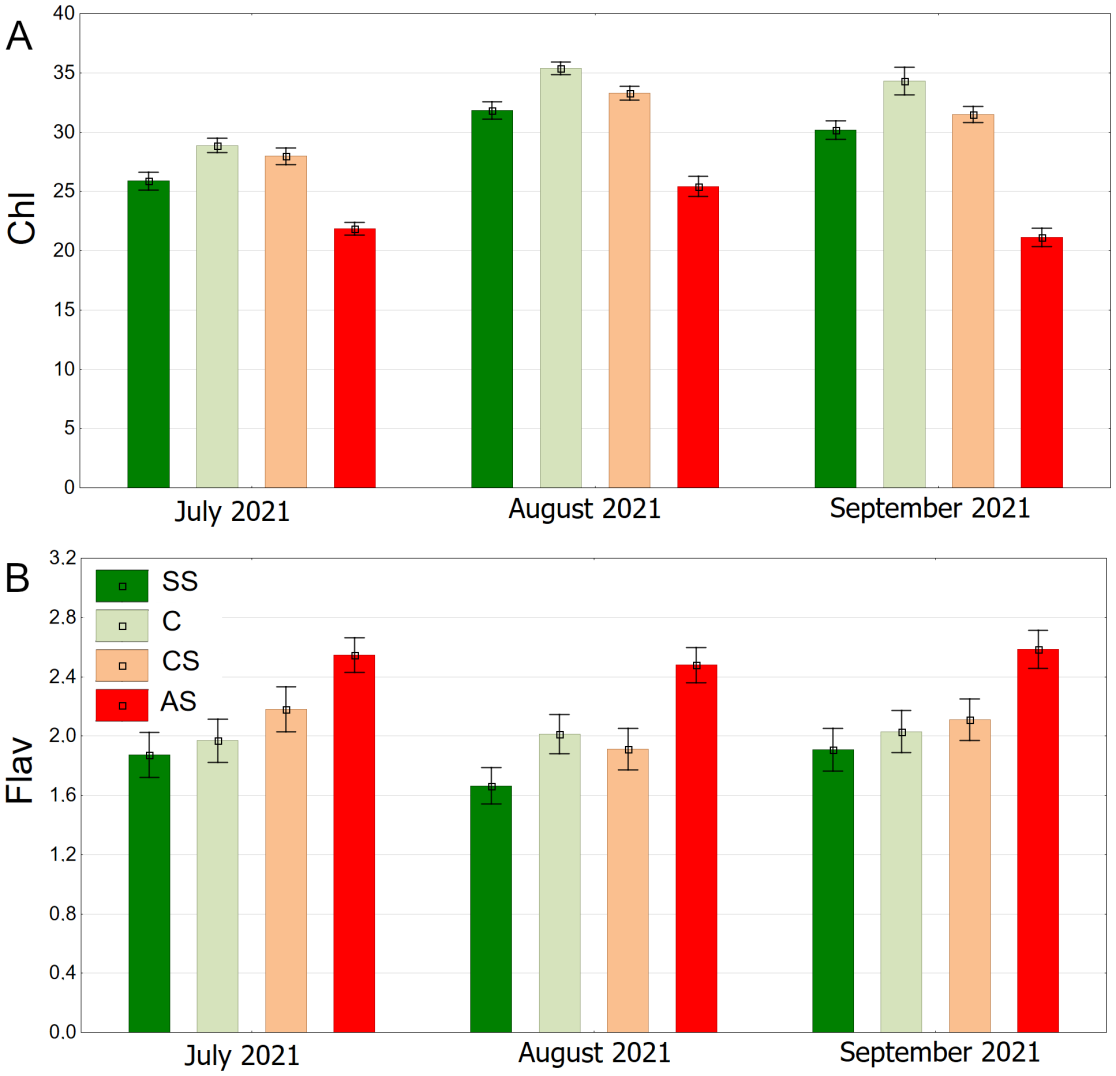
(A–B) Chlorophyll (Chl) and flavanols (Flav), results from all measurement dates. SS, structural soil; C, control; CS, compacted soil; AS, impermeable paving. Bars show means and SE.

The distribution of chlorophyll content is clearly seasonal. The overall trend shows higher values in August, and lower values in July and September. On each of the three dates, the highest chlorophyll levels were recorded for the control plot (C), then CS, SS, and the lowest AS (Fig. 14A). The highest flavonoid values were in the AS sector. The lowest flavonoid levels were observed in SS and C. Flavonoid values remained relatively stable throughout the study period (Fig. 14B).

## Discussion

The results obtained in the initial period of *Tilia tomentosa* establishment in our experimental site showed that structural soil provides optimal conditions for the *F*_v_/*F*_m_ parameter, PI_ABS_, flavanols content and higher root growth (Kociel, 2018). However, trees are organisms of a long lifespan, and it is necessary to provide them with appropriate living conditions for a several dozen of years. In our experiment, carried out 5 and 6 years after planting the plants, trees that grew under site conditions simulating urban conditions (CS) and harsh urban conditions combining high soil compaction with applied impervious surfaces (AS) showed decreased *F*_v_/*F*_m_, which is a key index of photosynthetic light conversion into biochemical energy, indicating a weakened physiological condition of trees growing in CS and AS. As photosynthetic activity provides energy for plant growth and stress recovery, the low photosynthetic efficiency is often combined with limited tree growth (Swoczyna et al., 2014; Guo et al., 2016).

In our study the physiological parameters of the trees growing in SS did not differ significantly from the parameters of the trees growing in natural soil. Grabosky & Bassuk (2008) found that after a ten-year period trees growing in paved soil (planted in structural soil covered with concrete and paving stones) had dendrometrical parameters comparable to the parameters of the trees growing on the lawn section of an experimental plot (specifically when it comes to their overall health, height gain, and trunk diameter). We have observed the same phenomenon when analyzing the physiological reactions of the trees we studied. Environmental stress causes disturbances in the functioning of the photosynthetic apparatus of plants (Callowa, May & Johnstone, 2018). *F*_v_/*F*_m_ of the studied trees growing in a mixture of stony soils did not differ significantly from trees growing in natural soil without soil interference. For the other two physiological parameters the application of structural soil was similar to those obtained for trees growing in compacted soil conditions. Samples obtained from the trees growing in the structural soil and the control achieved the highest values of the *F*_v_/*F*_m_ ratio. Almost all the measurements in these groups were within the values of 0.78–0.85, which indicates their good health in deciduous trees (Percival, 2005; Callowa, May & Johnstone, 2018). Lower values were shown in samples collected from trees growing in the compacted soil and the impermeable paving. Similarly, Moore, Fitzgerald & May (2019) found that soil compaction affects the *F*_v_/*F*_m_ value. In the case of AS, the trees’ *F*_v_/*F*_m_ decreased to 0.6, indicating a disruption of PSII (Kalaji & Guo, 2008). In our experiment the similar *F*_v_/*F*_m_ values in trees growing in SS and C suggest that structural soils provide habitat condition appropriate to maintain *F*_v_/*F*_m_ at a satisfying level. The undisturbed process of energy conversion in PSII reaction centres was also reflected by the energy dissipation per RC (DI_0_/RC), which was not significantly different in SS and C trees except for September 2022. The RE_0_/ET_0_ parameter describing the reduction of end electron acceptors at PSI was the least dependent on tree growth conditions. Thus, we suppose that the reaction centres of both photosystem II and photosystem I are not susceptible to artificially composed soil as long as such soil meets the minimum conditions necessary to avoid compaction, which negatively affects photosynthesis (Asif et al., 2023).

The lower efficiency of electron donation from OEC in comparison to control, reflected by higher values of V_K_/V_J_, indicates that both the soil compaction and structural soil affect the donor side of photosystem II negatively. The susceptibility of the oxygen evolving complex is usually bound to thermal stress (Duan et al., 2015) or to drought stress (Kalaji et al., 2018). In urban environments V_K_/V_J_, denoted by some authors as W_K_ or W_300_, allows to detect difficulties in habitats where trees grow (Ugolini et al., 2012). In our experiment, higher values of V_K_/V_J_ suggest that structural substrates may trigger some disturbances in photosynthetic efficiency but usually not as much as heavily transformed urban soils.

The parameter describing the fate of the energy transfer between two photosystems, *i.e.,* on the biochemical pathway in the electron transport chain, ET_0_/TR_0_ showed some decline in trees growing in SS compared to trees growing in control. However, the SS results were mostly similar to CS. The ET_0_/TR_0_ decrease compared to control was reflected by ΔV_IP_ and PI_ABS_. These two parameters are calculated on the basis of ET_0_/TR_0_ (Strasser, Tsimilli-Michael & Srivastava, 2004; Strasser et al., 2010; Bussotti et al., 2020). On the other hand, the ET_0_/RC, reflecting the electron transport flux per active RC, showed significantly higher values in SS than control but similar to CS and AS (Figs. 9B, 9E). This fact may be explained by the lower density of active reaction centres per cross section (RC/CS_0_) in stressed trees. The fewer the active reaction centres, the less loaded electron carriers are present in the electron transport chain. However, this parameter may show the plants’ ability to overcome photoinhibition stress. For example, Mlinarić et al. (2017) found higher ET_0_/RC values in *Ficus carica* mature leaves than in the younger ones, which are more susceptible to photoinhibition at noon hours. ET_0_/RC also positively correlated with winter survival in herbs growing in a vertical gardens (Swoczyna et al., 2020).

The decrease of RC/CS_0_ may result from drought stress (Lee et al., 2016), when the deficit of water leads to the inactivation of some parts of the PSII reaction centres (Guha, Sengupta & Reddy, 2013). However, low values of relative chlorophyll obtained from the chlorophyll meter indicate that the diminished chlorophyll content in leaves could affect the number of RCs (Castro et al., 2011). The relation between leaf nitrogen content and chlorophyll content has been found by Loh, Grabosky & Bassuk (2002) and Cartelat et al. (2005) as well. The density of reaction centres per cross section highly depends on the leaf nitrogen content and nitrogen nutrition (Swoczyna et al., 2019). In natural site conditions, the nitrogen for the plants comes from soil organic matter. In artificially created structural soils, the lack of organic matter may lead to nitrogen deficiency in the trees. The lowest chlorophyll content in the substructure of the pavement designed as a substrate with an impermeable pavement indicates the particularly difficult habitat for trees in such designs. The lower values of PI_ABS_ in the impervious pavement, compacted and structural soils also indicate that these three types of soils do not provide the same growth conditions as natural soil. However, Performance Index is a parameter which also integrates the rate of reaction centres, thus, the pattern of differences between examined plots may be similar to the pattern of RC/CS_0_ differences. In September 2021 PI_ABS_ values decreased visibly comparing to previous months. Two other parameters, *F*_v_/*F*_m_ and ET_0_/TR_0_, are also included in the PI_ABS_ mathematical formula. Therefore, the PI_ABS_ values from September 2021 were likely reduced by the decreased *F*_v_/*F*_m_ and ET_0_/TR_0_.

The statistically significant higher content of flavanols in our study concerning impermeable paving samples confirms that such conditions are extremely harsh for trees. The lowest flavanol content results were recorded in the leaves of trees growing in structural soils. This indicates that these soils, while not providing conditions for optimal chlorophyll content, do not lead to increased stress in leaves which might induce higher accumulation of flavanols in leaves. Flavanols play an important role in plants, such as protection from abiotic stress. The increased accumulation of flavanols is often a response to UV-B radiation, drought, heat, ozone or salinity (Daryanavard et al., 2023). For this reason we used the flavanol content as an additional indicator of stress in our study. One of the roles of flavanols is to scavenge reactive oxygen species (ROS) which are accumulated in the face of disturbed biochemical processes. In turn, the purpose for accumulation of chlorophylls is to ensure production of sugars, while the reasons for limited chlorophyll accumulation are often different from those modulating flavanol accumulation, for example restricted N availability (Kalaji et al., 2017). Therefore, the stress response showed as poor accumulation of chlorophyll might not be reflected by the response showed as increased flavonols accumulation.

An experiment carried out on the same site two years after planting, indicated good vitality of trees growing in the structural soil plot and better metrics of some tested parameters compared to trees planted in different habitat conditions: natural soil, compacted substrate or extemporarily heavy (a mixture of soil and rubble covered with impermeable pavement) (Kociel et al., 2018). In that study the SS provided the best results of the *F*_v_/*F*_m_ parameter, PI_ABS_ and flavanol content. The worst results were recorded for trees growing in impermeable soils. In our study, the results are similar, but in the case of the PI_ABS_ index and chlorophyll content, only the control sample showed the most favourable parameters. On the other hand, in an experiment conducted by Kociel et al. (2018), the trees planted in structural soil showed higher root growth and had lower crown temperatures. The authors explained the values of those parameters using the higher transpiration intensity which stemmed from better access to water and the higher overall vitality of the trees.

Ordóñez Barona et al. (2018), found that 7 years after planting the trees, the salinity levels in the structural soil pavement subgrade elevated beyond those suggested before and reportedly caused negative effects for tree vitality, in addition to lower levels of soil salinity and alkalinity which correlated with better tree vitality. Salinity was excluded from our study (the experimental plot was protected) but SS was designed with the consideration of delivering proper pH levels for root development to avoid alkalinity. Another study of urban soils in Boston and Massachusetts included sand-based structural soils, rock-based structural soils, and a horticultural mix, tested between 6 and 45 years after establishment. The results show that the pedogenic processes occur in these soils at different rates and magnitudes. The illuviation of clay, acidification, and a decrease in the mineralization quotient were observed over time. This study shows that SS are not static systems and the way the systems are taken care of should be adapted to the soil changes occurring over time (Scharenbroch et al., 2018). In our experiment we examined the trees, 5 and 6 years following their planting, *i.e.,* after the crucial time of the 3 years necessary for the acclimation at a new site (Gerhold & McElroy, 1994).

In a study of the long-term response of trees to the application of structured substrate in the U.S., after a 17 year period of observation, better conditions were observed, and a larger crown size and intensely green leaf blade colour were recorded in comparison to trees growing on lawns (Bassuk et al., 2015). No visual differences in leaf colour intensity were observed in our study. However, the lower relative chlorophyll content and RC/CS_0_ value suggests that attention should be paid in the future to improving nitrogen availability in the root zone of trees planted in structural substrates. On the other hand, despite being covered with a permeable resin concrete pavement, the conditions for photosynthetic functioning of these trees were more favourable than for a mixture of soil and rubble with a concrete slab pavement.

Our results explain the findings of other authors which indicate that urban conditions, *i.e.,* soil compaction and limited space for roots development (Jim, 2023), limited water infiltration and air-gas exchange into the soil (Kostić et al., 2019), and drought (Kostić et al., 2022), are associated with reduced growth, poorer health and shallow root colonization (Bartens et al., 2009; Embrén et al., 2009; Grabosky et al., 2001; Grabosky & Bassuk, 2016). SS allows the support of all the factors mentioned above (oxygen and water availability for tree roots under pavements) which have a significant potential to improve site conditions, unfortunately SS is still underestimated and thus consequently—not applied in numerous areas in which it is needed.

It is important to acknowledge the limitations of this study, particularly the lack of replication at the experimental block level. Therefore, future research should be conducted across multiple urban locations. Such an expansion would enable the collection of a more extensive data set.

## Conclusions

The study confirms the postulated hypothesis that structural substrate provides a significant improvement in habitat conditions for tree development in urban environments over a multi-year period. Considering all the bioindicators studied, the leaves obtained from the impermeable paving plot showed the weakest parameters reflecting poor plant health. On the contrary, structural soil revealed satisfying conditions for PSII functionality compared to “urban soils”, compacted soils and impermeable pavement. Trees in structural soil were in good condition, and their health parameters did not deteriorate significantly compared to trees from the control group. We have shown that structural soil provides satisfying conditions for photosynthetic processes in trees in the 6 years after planting, which confirms their applicability as a solution in site conditions exposed to soil compaction or as a solution for enlarging root pits with spaces under pavements. The insufficient availability of nutrients, especially nitrogen, may be a problem in the future. Therefore, further investigation in this direction is needed.

Structural substrate has the potential to maintain the vitality of urban trees growing in harsh conditions. Structural substrates, due to their specificity, can be used as an alternative solution for providing proper site conditions for trees in urban environments due to their potential to extend the tree lifespan. Further research should be developed in the field of the proper application of SS (*i.e.,* moderate drainage conditions). Structural substrates may provide an additional possibility to implement tree planting in particularly difficult sites and this way enhance the potential for adaptation to climate change in urbanized areas.

##  Supplemental Information

10.7717/peerj.20407/supp-1 Supplemental Information 1 Dane surowe: Chl and Flav

10.7717/peerj.20407/supp-2 Supplemental Information 2 Raw data: fluorescence of chlorophyl

## Additional Information and Declarations

### Data Availability

The following information was supplied regarding data availability: The raw measurements are available in the Supplemental Files.
